# Effects of time of the day at sampling on CSF and plasma levels of Alzheimer’ disease biomarkers

**DOI:** 10.1186/s13195-024-01503-x

**Published:** 2024-06-22

**Authors:** Anna Orduña Dolado, Erik Stomrud, Nicholas J. Ashton, Johanna Nilsson, Clara Quijano-Rubio, Alexander Jethwa, Wagner S. Brum, Ann Brinkmalm Westman, Henrik Zetterberg, Kaj Blennow, Shorena Janelidze, Oskar Hansson

**Affiliations:** 1 Clinical Memory Research Unit, Department of Clinical Sciences Malmö, Faculty of Medicine, Lund University, Sölvegatan 19, BMC B11, Lund, 221 84 Sweden; 2 Memory Clinic, Skåne University Hospital, S:t Johannesgatan 8, Malmö, SE-20502 Sweden; 3 Department of Psychiatry and Neurochemistry, Institute of Neuroscience and Physiology, the Sahlgrenska Academy at the University of Gothenburg, Mölndal, Sweden; 4 Centre for Age-Related Medicine, Stavanger University Hospital, Stavanger, Norway; 5 Department of Old Age Psychiatry, Maurice Wohl Clinical Neuroscience Institute, King’s College London, London, UK; 6 NIHR Biomedical Research Centre for Mental Health & Biomedical Research Unit for Dementia at South London & Maudsley NHS Foundation, London, UK; 7 Clinical Neurochemistry Laboratory, Sahlgrenska University Hospital, Mölndal, Sweden; 8 Roche Diagnostics International Ltd, Rotkreuz, Switzerland; 9 Roche Diagnostics GmbH, Penzberg, Germany; 10 Graduate Program in Biological Sciences: Biochemistry, Universidade Federal do Rio Grande do Sul (UFRGS), Porto Alegre, Brazil; 11 Department of Neurodegenerative Disease, UCL Institute of Neurology, Queen Square, London, UK; 12 UK Dementia Research Institute at UCL, London, UK; 13 Hong Kong Center for Neurodegenerative Diseases, Hong Kong, Hong Kong SAR China; 14 Wisconsin Alzheimer’s Disease Research Center, School of Medicine and Public Health, University of Wisconsin, University of Wisconsin-Madison, Madison, WI USA; 15 Pitié-Salpêtrière Hospital, Paris Brain Institute, ICM, Sorbonne University, Paris, France; 16 Neurodegenerative Disorder Research Center, Division of Life Sciences and Medicine, Department of Neurology, Institute on Aging and Brain Disorders, University of Science and Technology of China and First Affiliated Hospital of USTC, Hefei, P.R. China

**Keywords:** Alzheimer’s disease, Fluid biomarkers, p-tau, Aβ, Sampling time, Diurnal variability

## Abstract

**Background:**

Studies suggest that cerebrospinal fluid (CSF) levels of amyloid-β (Aβ)42 and Aβ40 present a circadian rhythm. However sustained sampling of large volumes of CSF with indwelling intrathecal catheters used in most of these studies might have affected CSF dynamics and thereby confounded the observed fluctuations in the biomarker levels.

**Methods:**

We included 38 individuals with either normal (N = 20) or abnormal (N = 18) CSF Aβ42/Aβ40 levels at baseline. CSF and plasma were collected at two visits separated by an average of 53 days with lumbar punctures and venipunctures performed either in the morning or evening. At the first visit, sample collection was performed in the morning for 17 participants and the order was reversed for the remaining 21 participants. CSF and plasma samples were analyzed for Alzheimer’ disease (AD) biomarkers, including Aβ42, Aβ40, GFAP, NfL p-tau181, p-tau217, p-tau231 and t-tau. CSF samples were also tested using mass spectrometry for 22 synaptic and endo-lysosomal proteins.

**Results:**

CSF Aβ42 (mean difference [MD], 0.21 ng/mL; *p* = 0.038), CSF Aβ40 (MD, 1.85 ng/mL; *p* < 0.001), plasma Aβ42 (MD, 1.65 pg/mL; *p* = 0.002) and plasma Aβ40 (MD, 0.01 ng/mL, *p* = 0.002) were increased by 4.2-17.0% in evening compared with morning samples. Further, CSF levels of 14 synaptic and endo-lysosomal proteins, including neurogranin and neuronal pentraxin-1, were increased by 4.5-13.3% in the evening samples (MD_range_, 0.02-0.56 fmol/µl; *p* < 0.042). However, no significant differences were found between morning and evening levels for the Aβ42/Aβ40 ratio, different p-tau variants, GFAP and NfL. There were no significant interaction between sampling time and Aβ status for any of the biomarkers, except that CSF t-tau was increased (by 5.74%) in the evening samples compared to the morning samples in Aβ-positive (MD, 16.46 ng/ml; *p* = 0.009) but not Aβ-negative participants (MD, 1.89 ng/ml; *p* = 0.47). There were no significant interactions between sampling time and order in which samples were obtained.

**Discussion:**

Our findings provide evidence for diurnal fluctuations in Aβ peptide levels, both in CSF and plasma, while CSF and plasma p-tau, GFAP and NfL were unaffected. Importantly, Aβ42/Aβ40 ratio remained unaltered, suggesting that it is more suitable for implementation in clinical workup than individual Aβ peptides. Additionally, we show that CSF levels of many synaptic and endo-lysosomal proteins presented a diurnal rhythm, implying a build-up of neuronal activity markers during the day. These results will guide the development of unified sample collection procedures to avoid effects of diurnal variation for future implementation of AD biomarkers in clinical practice and drug trials.

**Supplementary Information:**

The online version contains supplementary material available at 10.1186/s13195-024-01503-x.

## Introduction

There is a great need for fluid biomarkers that robustly reflect various aspects of the pathophysiology of Alzheimer’s disease (AD) to improve the diagnostic workup, monitor progression and enable effective drug-development. Currently available fluid biomarkers include amyloid-β (Aβ)42, alone or in ratio with Aβ40 and phosphorylated tau (e.g., p-tau181, p-tau217 and p-tau231) reflecting core AD-related Aβ and tau pathologies, respectively. Additional promising biomarkers of pathophysiological processes that are common for many neurodegenerative disorders are neurofilament light (NfL), a marker of axonal degeneration, as well as a marker of glial activation, glial fibrillary acidic protein (GFAP) [1]. Although cerebrospinal fluid (CSF) measures are available and have proven highly useful as diagnostic and prognostic tools for AD in research settings, clinical care and drug trials, blood testing offers several advantages (e.g., lower invasiveness, higher accessibility, cost-effectiveness) [2]. Accumulating evidence suggests that plasma Aβ42/40, different p-tau isoforms (p-tau181, p-tau231 and p-tau217), NfL and GFAP approach in performance [3–9] or even outperform [10] their corresponding CSF biomarker.

To improve biomarker performance in clinical care and trials, it is important to implement standardized sample collection and handling procedures that would minimize the effects of pre-analytical component among factors impacting biomarker variability [11–14]. One such pre-analytical factor to consider is time of the day at sample collection. Even though, published protocols for CSF sampling recommend to perform lumbar puncture (LP) at a standardized time (08.00–12.00 AM) to avoid potential diurnal variation for CSF biomarkers [15], diurnal variability in CSF and plasma concentrations of AD biomarkers is not well established. Early reports showed fluctuations of CSF Aβ with 1.6-to-4-fold change over a 36-hour period in younger non-demented participants with good general health [16]. Other studies have found smaller (5.5 to 6.7%) or no significant fluctuation in older, more clinically relevant cohorts [17, 18]. Two studies have investigated Aβ dynamics in plasma, reporting 5–9% higher levels of Aβ42 and Aβ40 in samples collected in the afternoon versus morning and larger diurnal fluctuations in younger individuals than in older individuals [19, 20]. While data on NfL and GFAP, either in CSF or plasma are lacking, some evidence suggest that CSF t-tau or p-tau levels do not follow diurnal pattern both in healthy old population [17] and in neurosurgical patients with CSF pressure monitoring [21].

Synaptic homeostasis alteration and degeneration are early pathological events common in many neurodegenerative diseases, including AD. This makes synaptic proteins that reflect synaptic dysfunction interesting early biomarkers [22]. Disruption of sleep and circadian rhythm is believed to happen with ageing and contribute to development of neurodegenerative diseases in part through synaptic dysfunction [23]. AD as well as other proteinopathies are also accompanied by aberrant function of endo-lysosomal networks [24]. Several articles reported increased levels of endo-lysosomal proteins in CSF of patients with AD while their levels seem to decrease in Parkinson’s disease [25–28]. To the best of our knowledge, no studies have assessed variations in synaptic or endo-lysosomal protein levels during the day in humans.

Most studies on changes in CSF Aβ levels to date used frequent and sustained sampling throughout the day with an indwelling intrathecal catheter. This procedure has been shown to contribute to the rise in CSF Aβ42 and Aβ40 independent of circadian fluctuations as repeated lumbar sampling presumably drives the redistribution of CSF flow towards the lumbar space where it is collected [19, 29, 30]. To minimize the effect of sampling procedures on CSF biomarker concentrations, participants in the present study underwent two LPs, one in the morning and another one in the evening, separated by an average of 53 days and samples were analyzed for all major AD biomarkers as well as a panel of 22 synaptic and endo-lysosomal proteins. To ensure that changes in biomarker levels were not due to the sampling order, 17 participants had the first visit in the morning and the second in the evening and the order was reversed for the remaining 21 participants. In addition to CSF, we collected plasma samples on the same visit and quantified the most promising plasma AD biomarkers using currently best performing immunoassays [31, 32]. Our primary research question was whether the time at sample collection, morning or evening, affected the levels of different biomarkers (Aβ42, Aβ40, Aβ42/40, NfL, GFAP, p-tau217, p-tau181, p-tau231, t-tau and synaptic and endo-lysosomal proteins). A secondary research question was whether any of these differences were affected by the amyloid status of the participants.

## Methods

### Participants

Participants were enrolled at the Memory Clinic, Skåne University Hospital comprising clinical patients who underwent LP as a component of their clinical assessment, along with individuals from the longitudinal Swedish BioFINDER study. The inclusion of the participants from the BioFINDER study contributed to the relatively high numbers of asymptomatic subjects with unimpaired cognition. Participants were selected such that the numbers of Aβ + and Aβ- individuals were approximately the same. The sole inclusion criterion for participation in this study was the performance of a LP at the clinic. Exclusion criteria consisted of individuals who did not undergo the requisite two LP. All participants had two visits when LPs and venipunctures were performed approximately at the same time. We believe that any damage and CSF leakage caused by LP at the first visit would have healed after approximately one month. Therefore, study participants had second visit with LP and venipuncture on an average 53 days (range 41–65 days) after the first visit. For 17 participants the first collection was performed in the morning and the following in the evening and for the remaining 21 participants the order was reversed. Time difference between morning and evening samplings was on an average 10:30 h (range 9:45 − 11:45 h).

### Plasma and CSF collection and analysis

20mL of CSF was collected in 5-mL LoBind tubes. CSF was centrifuged (2000 g, + 4 °C) for 10 min, aliquoted in 1.5 mL polypropylene tubes and stored at − 80 °C within 30–60 min of collection [15]. Blood was collected in EDTA-plasma tubes (Vacutainer K2EDTA tube, BD Diagnostics) and centrifuged (2000 g, + 4 °C) for 10 min. Resulting plasma was transferred into one 50-mL polypropylene tube, mixed and aliquoted into 1.5 mL polypropylene tubes and stored at − 80 °C within 30–60 min of collection. All samples from the same patient were measured in the same run to limit the effects of run-to-run variability on biomarker concentrations.

CSF levels of Aβ40, t-tau, NfL and GFAP were measured as part of robust prototype assay within the NeuroToolKit, on fully automated cobas® e 411 or e 601 analyzers (all Roche Diagnostics International Ltd, Rotkreuz, Switzerland) as previously described [33]. CSF Aβ42 levels were measured as part of the Roche NeuroToolKit using the in vitro diagnostic (IVD) Elecsys® assay [34]. Plasma levels of Aβ42, Aβ40, GFAP and NfL were also measured as part of the Roche NeuroToolKit using Elecsys® plasma prototype immunoassays (All Roche Diagnostics International Ltd, Rotkreuz, Switzerland) on cobas® e 411 and cobas e 601 instruments as previously described [33]. CSF and plasma p-tau231 and p-tau181 levels were measured by an in-house Simoa assay developed in the University of Gothenburg, as previously described [35, 36]. CSF and plasma p-tau-217 levels were measured using an immunoassay developed by Lilly Research laboratories on the Meso-Scale Discovery Platform as previously described [4].

CSF samples were analyzed for a panel of 18 synaptic proteins and 4 endo-lysosomal proteins (See Supplementary Table 1, Supplemental 1) using liquid chromatography with tandem mass spectrometric analysis (LC–MS/MS) as previously described [37].

Study participants were classified as amyloid negative (Aβ-) or positive (Aβ+) using CSF Aβ42/Aβ40 quantified with the Food and Drug administration (FDA)-approved Lumipulse G assay and established cut-off of 0.072 [38].

### Statistical analyses

Differences in the demographic variables were evaluated with Student t-test (age, Mini Mental State Examination (MMSE) scores, estimated glomerular filtration rate (eGFR, as an indicator of kidney dysfunction) and Body Mass Index (BMI)) or Fisher’s exact test (gender, *APOE* ε4 carriership and diagnosis). Repeated measures two-way ANOVA including interaction effect between Aβ status and time at sampling was used to assess whether biomarker levels in Aβ + and Aβ- individuals were affected differently by time of sample collection. Similar analysis was carried out to assess interaction between order in which samples were collected (i.e., morning collection or evening collection first) and time at sampling. Multiplicity correction was applied using the Bonferroni-Dunn method except the CSF synaptic and endo-lysosomal panel where we used Benjamini–Hochberg false discovery rate (FDR). All significance were two-sided with significance level equal to 0.05. Statistical analysis was performed using Prism 9 (GraphPad Software, San Diego, California, USA).

## Results

### Participant demographics

The demographic and clinical data for all participants are summarized in Table 1. Out of 38 participants, 18 were Aβ-positive (Aβ+) and 20 *A*β-negative (Aβ-). There were no significant differences between Aβ + and Aβ- groups for sex, age, MMSE score, diagnosis, eGFR or BMI. There was a higher proportion of *APOE* ε4 carriership in Aβ + in comparison with Aβ- (70.6% vs. 15.0%, *p* = 0.002). Most study participants (35 out of 38) were cognitively unimpaired while 3 individuals were cognitively impaired.

**Table 1:** Demographics and clinical characteristics of all subjects

	All subjects	Aβ +	Aβ -	*P*-value Aβ^+^ vs. Aβ ^−^
N	38	18	20	
Age (years)	77 (5.91)	78 (4.41)	76 (6.86)	0.08
Gender (F/M)	16/22	8/10	8/12	0.52
*APOE* ε4 positivity, n (%)	15 (39)	12 (71)	3 (15)	**0.002**
MMSE	28 (2.10)	28 (1.84)	29 (2.25)	0.16
Diagnosis				
*Cognitively Unimpaired* *Cognitively Impaired*	35 3	16 2	19 1	0.32
Kidney Function (eGFR)^a^	122.29 (21.54)	119.71 (20.52)	124.57 (23.09)	0.54
BMI^b^	26.64 (2.84)	25.86 (2.31)	27.29 (3.13)	0.31

Data shown as mean (SD) unless specified otherwise

Abbreviations: M, male; F, female; *APOE*, apolipoprotein; MMSE, Mini-Mental State Examination, eGFR, estimated glomerular filtration rate; BMI, Body Mass Index

^a^ Data was missing in 5 participants (2 Aβ + and 3 Aβ -)

^b^ Data was missing in 3 participants (2 Aβ + and 1 Aβ-)

### CSF and plasma AD biomarkers

**Fig. 1:**
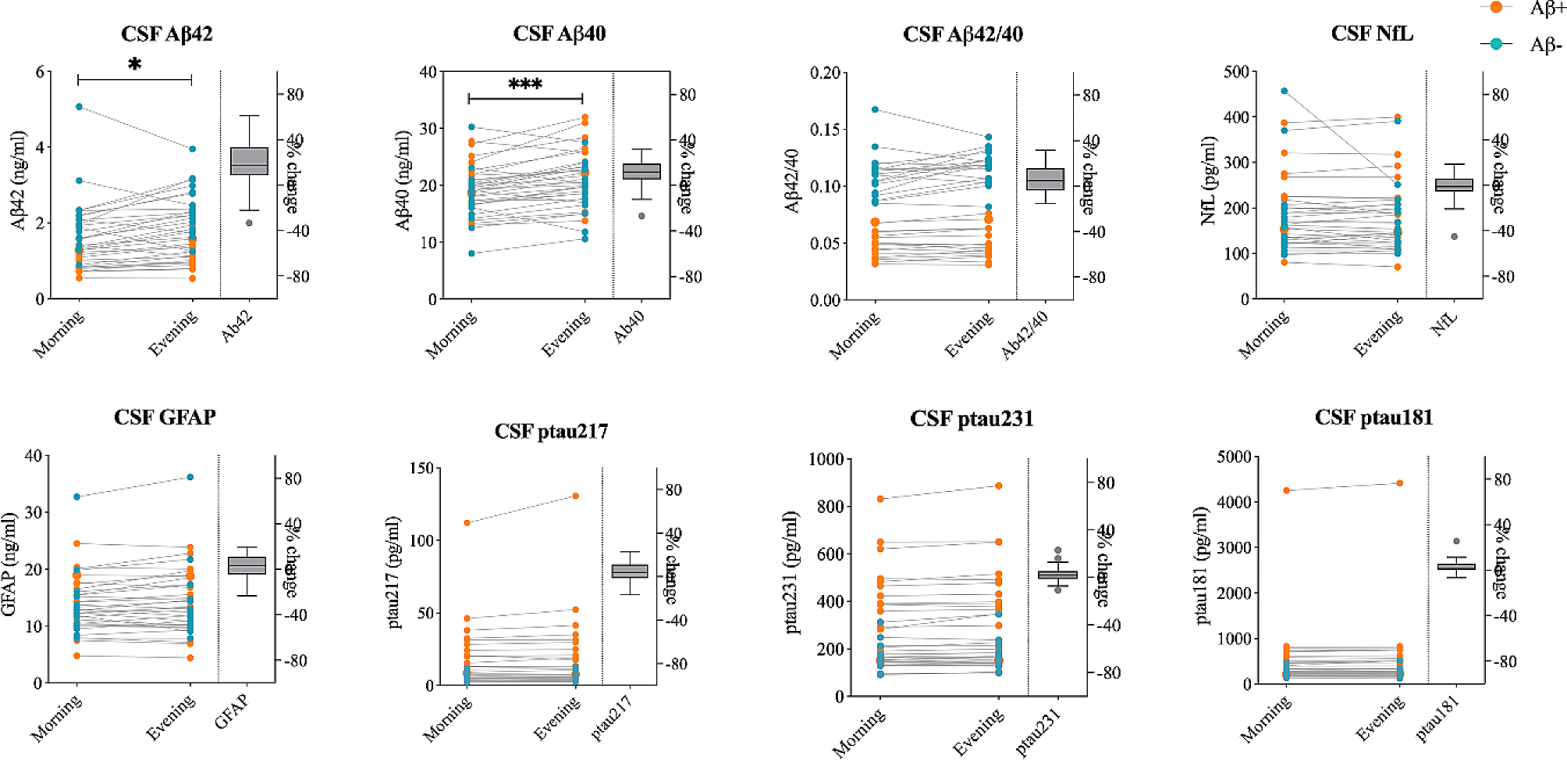
**CSF biomarkers levels in samples collected in the morning and evening**. Subject specific biomarker concentration in samples collected in the morning vs. evening. Average percent changes between time points are shown in box-plots plotted with the Tukey method. Blue and orange dots represent participants with negative and positive amyloid status, respectively. Asterisks represent *p*-values for the main effects of sampling time from repeated measures two-way ANOVA; * *p* < 0.05, *** *p* < 0.001

**Fig. 2:**
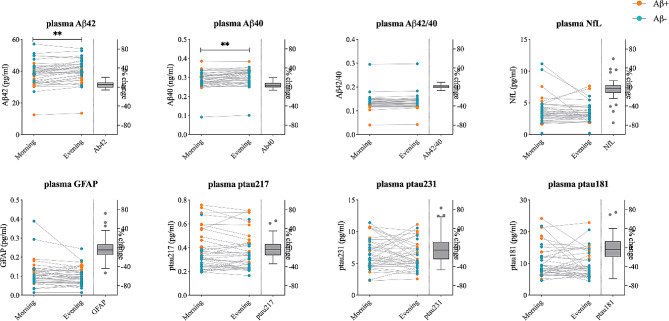
**Plasma biomarkers levels in samples collected in the morning and evening.** Subject specific biomarker concentration in samples collected in the morning vs. evening. Average percent changes between time points are shown in box-plots plotted with the Tukey method. Blue and orange dots represent participants with negative and positive amyloid status, respectively. Asterisks represent *p*-values for the main effects of sampling time from repeated measures two-way ANOVA; ** *p* < 0.01

CSF concentrations of Aβ42 (mean difference [MD], 0.21 ng/mL; *p* = 0.038) and Aβ40 (MD, 1.85 ng/mL; *p* < 0.001) were increased by 17.0% (95% CI, 10-24.1) and 10.5% (95% CI, 6.5–14.4), respectively, in samples collected in the evening compared to those collected in the morning (Fig. 1; Table 2). Similarly, plasma levels of Aβ42 (MD, 1.65 pg/mL; *p* = 0.002) and Aβ40 (MD, 0.01 ng/mL, *p* = 0.002) were significantly higher in samples collected in the evening compared to those collected in the morning. However, the increases were smaller for plasma Aβ42 (4.8%; 95% CI, 2.7–6.7) and Aβ40 (4.2%; 95% CI, 2.2–6.3) compared with CSF Aβ42 and Aβ40 (Fig. 2; Table 3). In contrast, we did not find any significant differences in either the CSF Aβ42/Aβ40 ratio (5.3%; 95% CI, 1.6- 9.0; *p* = 0.16) or the plasma Aβ42/Aβ40 ratio (0.7%; 95% CI, -0.7 to 1.1; *p* = 1.0) (Figs. 1 and 2; Tables 2 and 3). We also did not find any changes in CSF and plasma levels of p-tau217, p-tau181, p-tau231, NfL and GFAP (*p* = 1.00) between collection in the morning and evening (Figs. 1 and 2; Tables 2 and 3).

**Table 2:** CSF biomarker concentrations in samples collected in the morning and evening

Biomarkers	Morning concentration	Evening concentration	Differences evening-morning	% Change	F (df1, df2) ^a^	*P*-value corrected (uncorrected) ^b^
Aβ42 [ng/mL]	1.53 (0.85)	1.74 (0.82)	0.21 (0.35 to 0.07)	17.02 (9.99 to 24.06)	8.99 (1, 37)	**0.038** (0.005)
Aβ40 [ng/mL]	19.08 (4.44)	20.94 (4.86)	1.85 (1.09 to 2.62)	10.46 (6.50 to 14.42)	24.10 (1, 37)	**0.0008** (0.0001)
Aβ42/Aβ40 ratio	0.081 (0.03)	0.085 (0.04)	0.004 (0.001 to 0.008)	5.29 (1.57 to 9.01)	5.91 (1, 37)	0.16 (0.02)
P-tau217 [pg/mL]	14.50 (19.66)	15.52 (22.61)	1.02 (-0.06 to 2.10)	4.03 (0.81 to 7.25)	3.67 (1, 37)	0.50 (0.06)
P-tau231 [pg/mL]	269.54 (176.24)	272.73 (184.1)	7.84 (2.11 to 13.56)	2.60 (0.48 to 4.72)	7.71 (1, 36) ^c^	0.07 (0.009)
P-tau181 [pg/mL]	466.51 (669.2)	472.96 (687.58)	14.83 (3.76 to 25.90)	2.80 (0.94 to 4.66)	7.38 (1, 36) ^c^	0.08 (0.01)
GFAP [pg/mL]	13.76 (5.22)	14.25 (5.92)	0.49 (0.03 to 0.95)	2.59 (-0.64 to 5.83)	4.62 (1, 37)	0.30 (0.04)
NfL [ng/mL]	184.92 (84.22)	179.58 (76.04)	-5.33 (-6.52 to 17.18)	-1.54 (-5.19 to 2.12)	0.83 (1, 37)	1 (0.37)

Data shown as mean (SD) or mean (95%CI) unless specified otherwise. Abbreviations: CSF, cerebrospinal fluid; Aβ, amyloid beta; GFAP, glial fibrillary acidic protein; NfL, neurofilament light; P-tau, phosphorylated tau

^a^ Main effects of sampling time from repeated measures two-way ANOVA

^b^*P*-values were corrected for multiple comparison using the Bonferroni-Dunn method

^c^ Data was missing for 1 participant

**Table 3:** Plasma biomarker concentration in samples collected in the morning and evening

Plasma Biomarkers [units]	Morning concentration	Evening concentration	Differences evening-morning	% Change	F (df1, df2) ^a^	*P*-value corrected (uncorrected) ^b^
Aβ42 [pg/mL]	38.43 (7.76)	40.08 (7.37)	1.65 (0.84 to 2.47)	4.84 (2.68 to 7)	16.81 (1, 37)	**0.002 (0.0002)**
Aβ40 [ng/mL]	0.29 (0.05)	0.30 (0.05)	0.01 (0.006 to 0.02)	4.24 (2.15–6.32)	16.05 (1, 37)	**0.002 (0.0003)**
Aβ42/40	0.14 (0.03)	0.14 (0.03)	0.001 (-0.002 to 0.001)	0.65 (-0.70 to 1.10)	0.79 (1, 37)	1 (0.38)
P-tau217 [pg/mL]	0.37 (0.16)	0.36 (0.16)	-0.01 (-0.01 to 0.03)	-0.56 (-7.13 to 6.01)	0.95 (1, 37)	1 (0.34)
P-tau231 [pg/mL]	6.88 (3.05)	6.67 (3)	-0.22 (-0.52 to 0.95)	2.48 (-8.86 to 13.83)	0.35 (1, 35) ^c^	1 (0.55)
P-tau181 [pg/mL]	10.61 (4.89)	9.94 (4.43)	-0.71 (-0.76 to 2.10)	-0.15 (-11.75 to 11.46)	0.90 (1, 37)	1 (0.35)
GFAP [pg/mL]	0.11 (0.07)	0.10 (0.05)	-0.01 (-0.004 to 0.02)	-1.81 (-10.03 to 6.40)	2.04 (1, 37)	1 (0.16)
NfL [ng/mL]	3.83 (2.10)	3.42 (1.47)	-0.41 (-0.12 to 0.94)	-3.96 (-11.75 to 3.82)	2.46 (1, 37)	1 (0.13)

Data shown as mean (SD) or mean (95%CI) unless specified otherwise. Abbreviations: Aβ, amyloid beta; GFAP, glial fibrillary acidic protein; NfL, neurofilament light; P-tau, phosphorylated tau

^a^ Main effects of sampling time from repeated measures two-way ANOVA

^b^*P*-values were corrected for multiple comparison using the Bonferroni-Dunn method

^c^ Data was missing for 2 participants

Effects of sampling time on CSF and plasma biomarkers were not different in the Aβ+ and Aβ- groups (p_range_ uncorrected 0.06–0.88 for interaction between sampling time and Aβ status, Supplementary Table 3, Supplementary Figs. 2–3), except CSF t-tau (*p* = 0.007). There was a relatively small increase (5.74% (95% CI, 2.85–8.63)) in CSF t-tau levels in evening samples compared to the morning samples in Aβ+ participants (MD, 16.46 ng/ml; *p* = 0.009) but not in Aβ- participants (1.20% (95%CI, -1.99-4.39); MD, 1.89 ng/ml; *p* = 0.47) (Supplementary Fig. 2).

Morning or evening biomarker levels were not different depending on the order in which samples were collected (morning first vs. evening first) for any of the biomarkers (p_range_ uncorrected 0.051–0.85 for interaction between sampling time and order of sample collection, Supplementary Table 3).

### Synaptic and endo-lysosomal panel

We found differences between morning and evening samples for 14 out of 22 synaptic and endo-lysosomal proteins (Table 4 and Supplementary Table 2, Supplemental 1). CSF levels of amyloid precursor protein (APP), syntaxin-1B (STX1B), neurogranin (Ng), neuronal pentraxin receptor (NPTXR), neuronal pentraxin 1 (NPTX1), β-synuclein (β-Syn), γ-synuclein (γ-Syn), 14-3-3ε, phosphatidylethanolamine-binding protein 1 (PEBP-1), cathepsin F (CTSF), GM2 activator (GM2A), neurosecretory protein VGF (VGF), secretogranin-2 (SgII) and chromogranin A (CgA) were all increased by 4.5-13.3% (95% CI, 1.7–7.2 to 4.4–22.1; *p* < 0.048) in samples collected in the evening compared to those collected in the morning (Supplementary Fig. 1, Supplemental 1, Table 4).

**Table 4:** CSF synaptic and endo-lysosomal biomarkers with increased levels in samples collected in the evening vs. morning

Biomarkers	Morning concentration (fmol/µl)	Evening concentration (fmol/µl)	Differences evening-morning	% Change	F (df1, df2)^a^	*P*-value corrected (uncorrected) ^b^
**Synaptic panel**						
Amyloid Precursor Protein	3.80 (1.32)	4.04 (1.41)	0.24 (0.12 to 0.37)	7.06 (4.02 to 10.10)	15.46 (1, 37)	**0.005 (0.0004)**
Syntaxin-1B	0.31 (0.10)	0.34 (0.11)	0.02 (0.01 to 0.03)	7.10 (3.38 to 10.81)	13.77 (1, 37)	**0.005 (0.0007)**
Neuronal pentraxin receptor	1.22 (0.55)	1.35 (0.60)	0.12 (0.05 to 0.19)	12.15 (6.15 to 18.15)	13.58 (1, 37)	**0.005 (0.0007)**
Neuronal pentraxin-1						
*ETVLQQK* *LTPGEVYNLATCSTK*	0.67 (0.24) 1.52 (0.46)	0.71 (0.26) 1.61 (0.49)	0.04 (0.01 to 0.07) 0.09 (0.04 to 0.14)	7.66 (3.28 to 12.04) 6.24 (3.02 to 9.47)	8.38 (1, 37) 11.92 (1, 37)	**0.020 (0.006)** **0.008 (0.001)**
Neurogranin	0.60 (0.28)	0.66 (0.31)	0.06 (0.02 to 0.10)	11.59 (6.64 to 16.54)	10.56 (1, 37)	**0.012 (0.003)**
β-synuclein	0.28 (0.10)	0.30 (0.12)	0.03 (0.04 to 0.007)	9.59 (3.90 to 15.28)	7.91 (1, 37)	**0.020 (0.008)**
γ-synuclein	0.60 (0.21)	0.64 (0.20)	0.13 (0.001 to 0.26)	8.50 (3.31 to 13.70)	5.82 (1, 37)	**0.042 (0.021)**
PEBP- 1	7.91 (2.43)	8.23 (2.57)	0.32 (0.10 to 0.54)	4.45 (1.71 to 7.20)	8.96 (1, 37)	**0.020 (0.005)**
14-3-3ε	0.43 (0.14)	0.45 (0.16)	0.03 (0.01 to 0.05)	6.62 (2.29 to 10.96)	8.05 (1, 37)	**0.020 (0.007)**
Chromogranin-A	4.04 (2.82)	4.38 (2.99)	0.34 (0.10 to 0.57)	9.38 (4.97 to 13.78)	8.2 (1, 37)	**0.020 (0.007)**
Secretogranin-2	4.10 (1.55)	4.36 (1.74)	0.26 (0.06 to 0.47)	7.06 (2.65–11.47)	6.75 (1, 37)	**0.030 (0.013)**
Neurosecretory protein VGF						
*NSEPQDEGELFQGVDPR* *AYQGVAAPFPK*	3.77 (2.00) 7.52 (4.02)	3.98 (2.13) 8.08 (4.49)	0.21 (0.03 to 0.38) 0.56 (0.14 to 0.98)	6.38 (1.89 to 10.88) 8.45 (3.47 to 13.43)	5.84 (1, 37) 7.41 (1, 37)	**0.041 (0.021)** **0.023 (0.010)**
**Endo-lysosomal panel**						
Ganglioside GM2 activator						
*EVAGLWIK* *ESVLSSSGK*	3.91 (1.45) 5.38 (1.95)	4.11 (1.49) 5.60 (2.01)	0.20 (0.10 to 0.30) 0.23 (0.11 to 0.35)	5.95 (2.94 to 8.96) 4.70 (2.27 to 7.13)	15.87 (1, 37) 14.15 (1, 37)	**0.005 (0.0003)** **0.005 (0.0006)**
Cathepsin F	0.45 (0.13)	0.49 (0.13)	0.04 (0.01 to 0.07)	13.29 (4.44 to 22.14)	7.97 (1, 37)	**0.020 (0.008)**

Data shown as mean (SD) or mean (95%CI) unless specified otherwise. Data for all synaptic and endo-lysosomal proteins is shown in Supplementary Table 2. Abbreviations: CSF cerebrospinal fluid

^a^ Main effects of sampling time from repeated measures two-way ANOVA

^b^*P*-values were corrected for multiple comparison using the FDR method

Effects of sampling time were not different in the Aβ + and Aβ- groups (p_range_ uncorrected 0.06–0.88, for interaction between sampling time and Aβ status) for any of the proteins from the MS panel. In addition, morning or evening protein levels were not different depending on the order in which samples were collected (morning first vs. evening first) (p_range_ uncorrected 0.09–0.85, for interaction between sampling time and order of sample collection).

## Discussion

In this study, we show higher levels of Aβ42 and Aβ40 in samples collected in the evening compared to those collected in the morning. The increases were modest and consistent in both CSF and plasma. Importantly, no changes were observed in the Aβ42/Aβ40 ratio, or any other tested AD biomarker (i.e., p-tau217, p-tau231, p-tau181, NfL and GFAP) either in CSF or plasma. Additionally, 14 out of 22 synaptic and endo-lysosomal proteins were also increased in CSF in the evening in comparison to the morning samples.

Although there have been handful of studies on diurnal variation in the CSF levels of AD biomarkers, results have been inconsistent. Some have pointed to fluctuations in biomarker concentrations during the day [16, 17, 30], whereas other have not found any significant changes [18, 21]. The conflicting results are possibly caused by the small sample size in several of the studies, cohort specific differences as well as differences in the CSF sampling methods and assays used for Aβ quantification. Many reports have highlighted that frequent sampling and extraction of large volumes of CSF via indwelling catheter leads to increased levels of CSF Aβ [17, 19, 29, 39–41] possibly by promoting the transfer from the interstitial fluid to CSF [17] and by redistribution of fluid towards the lumbar space [29, 41]. To mitigate these sampling-related effect, CSF in the present study was collected at two separate LPs with an average interval of 53 days allowing sufficient time for tissue damage caused by the LP to heal. Of note, no interaction effects were seen between sampling time (i.e., morning and evening) and order in which samples were collected for any biomarker indicating that the differences in levels we report were not due to samples being collected at a later date. Our results support those that suggest a circadian rhythm for Aβ42 and Aβ40, with higher levels in the evening. The increases in CSF were modest with 17% and 10.5% for Aβ42 and Aβ40, respectively, (Table 2) and in a similar range (3.8–15%) to some studies that have included elderly subjects as well as patients with AD [17, 29]. The increases in plasma Aβ42 and Aβ40 were lower than in CSF (4.8% and 4.2% respectively; Table 3) and in a similar range (2-9%) as in previous reports [19, 20]. The smaller changes in the evening of Aβ in plasma could be partly due to the contribution of peripheral sources of Aβ that are less affected by circadian rhythms. The differences in Aβ42 and Aβ40 levels in the morning and evening samples were not influenced by brain Aβ status, which is important since 47% of our sample had abnormal Aβ-status (Table 1). These findings are in line with previous reports indicating that in elderly individuals day/night variability in Aβ42 and Aβ40 levels, did not vary between Aβ + and Aβ- groups [19, 39]. At the same time, we show that CSF and plasma Aβ42/Aβ40 ratios remained unaltered, suggesting that increased production or decreased clearance of Aβ peptides during daytime similarly affect the CSF and plasma levels of the Aβ42 and Aβ40.

Our results with higher APP levels in the evening in comparison to the morning suggest that circadian rhythm and synaptic activity might affect brain and CSF Aβ levels through modulation of APP expression, release and/or metabolism (Table 4). Aβ42 and Aβ40 are produced by the cleavage of APP and increased synaptic activity promotes the amyloidogenic processing of APP [42] leading to increased interstitial Aβ levels [43]. Interestingly, it has been shown in mice that interstitial fluid levels of Aβ correlate with time spent awake and change in response to activation of orexin which is known to regulate wakefulness under physiological conditions and follow a diurnal fluctuation [44, 45].

In agreement with earlier data, we did not find any significant fluctuations over the day for CSF or plasma p-tau [18, 21]. Furthermore, we show for the first time, that there are no differences in CSF and plasma NfL and GFAP concentrations between samples collected in the morning and evening. Collectively, these results suggest that during daytime there is a specific increase in the CSF and plasma levels of Aβ proteins rather than a general build-up of AD biomarkers.

Higher neuronal activity and increased synaptic strength during wakefulness compared to sleep have been reported in mice and rats [46–48]. High synaptic activity is associated with increased production of synaptic proteins, especially proteins that regulate the secretory pathways [49]. Taken together these findings may explain the higher levels of synaptic proteins in evening samples in comparison to the morning seen in our study (See ﻿Supplementary Table 2, Supplemental 1, Table 4). It remains unclear why only some synaptic and endo-lysosomal proteins were selectively affected in our study. Future investigations should explore the underlying mechanisms behind these findings.

The strength of the current study is that we assessed a wide range of the established and candidate CSF and plasma AD biomarkers measured using state-of-the art assays. However, this study has limitations. The sample size was relatively small and determined based on previous studies examining the effects of diurnal variability on Aβ levels (no power calculations were performed). The difference in biomarker levels between the morning and evening samples, in plasma in particular, were also small with intra-individual variability potentially influencing these results. Future work in larger cohorts accounting for the effects of intra-individual variability in biomarker concentration are warranted. These studies should also assess the impact of diurnal variability on diagnostic performance of AD biomarkers.

## Conclusions

In summary, we demonstrate that Aβ42 and Aβ40 levels in CSF and plasma have diurnal fluctuations with higher levels in the evening. Previous data have indicated that Aβ42/Aβ40 ratio is less affected than Aβ42 alone by different AD non-specific factors and pathologies (e.g., pre-analytical sample handling, inter-individual variability in Aβ levels, subcortical injury) [50]. Here, we also show that CSF and plasma Aβ42/Aβ40 levels are not influenced by the timing of the sample collection further supporting the use of Aβ42/Aβ40 ratio over Aβ42 alone in the diagnostic workup of AD. While the CSF and plasma levels of p-tau variants, NfL and GFAP did not exhibit diurnal variability, CSF levels of many synaptic and endo-lysosomal proteins were increased in samples collected in the evening. These results suggest an increase and build-up of markers associated with neuronal activity during wakefulness. In addition, our data highlight the need to consider the effects of circadian rhythms on the CSF (and potentially plasma) levels of synaptic and endo-lysosomal proteins that are considered as candidate biomarkers of AD. Overall, the findings of the present study support the standardization of sample collection protocols for AD biomarker determination, with sampling at a specific time interval during the day.

### Electronic supplementary material

Below is the link to the electronic supplementary material.

**Supplementary Material 1**: Supplementary Table 1, Supplementary Table 2, Supplementary Fig. 1, Supplementary Table 3, Supplementary Fig. 2 and Supplementary Fig. 3.

## Data Availability

Anonymized data from the study will be shared upon request from a qualified academic investigator.

## Abbreviations

AD: Alzheimer’s Disease
Aβ: Amyloid-β
APP: Amyloid precursor protein
BMI: Body mass index
β-syn: Beta-synuclein
CTSF: Cathepsin F
CSF: Cerebrospinal fluid
CgA: Chromogranin A
FDA: Food and Drug administration
FDR: False discovery rate
eGFR: Estimated glomerular filtration rate
GFAP: Glial fibrillary acidic protein
GM2A: GM2 activator
IVD: In-vitro diagnostic
LC-MS/MS: Liquid chromatography with tandem mass spectrometry
LP: Lumbar puncture
MD: Mean difference
MS: Mass spectrometry
MMSE: Mini-Mental State Examination
NfL: Neurofilament light
Ng: Neurogranin:
NPTX1: Neuronal pentraxin 1
NPTXR: Neuronal pentraxin receptor
PEBP-1: Phosphatidylethanolamine-binding protein 1
p-tau: Phosphorylated- tau
SgII: Secretogranin II
STX1B: Syntaxin-1B
VGF: Neurosecretory protein VGF
