# Supplementary material for: Effects of time of the day at sampling on CSF and plasma levels of Alzheimer’ disease biomarkers
Source: Alzheimers Res Ther. 2024 Jun 22;16:132. doi: 10.1186/s13195-024-01503-x (PMC11193266; doi:10.1186/s13195-024-01503-x)
Supplement: Supplementary file 1 — Supplementary Material 1: Supplementary Table 1, Supplementary Table 2, Supplementary Fig. 1, Supplementary Table 3, Supplementary Fig. 2 and Supplementary Fig. 3. [file 13195_2024_1503_MOESM1_ESM.pdf]

Supplementary Table 1: Synaptic and endo-lysosomal proteins included in the MS panel

Protein Name	Gene ID	Accession	Length	Peptide	Coverage (%)
**Synaptic panel**					
Amyloid Precursor Protein	APP	P05067	770	VESLEQEAANER	1.56
Neurosecretory protein VGF	VGF	O15240	615	NSEPQDEGELFQGVDPR	2.76
				AYQGVAAPFPK	1.79
Secretogranin-2	SCG2	P13521	617	ALEYIENLR	1.46
				VLEYLNQEK	1.46
Chromogranin-A	CHGA	P10645	457	GLSAEPGWQAK	2.41
				EDSLEAGLPLQVR	3.17
Rab GDP dissociation inhibitor alpha	GDIA	P31150	447	QLICDPSYIPDR	2.68
Phosphatidylethanolamine-binding protein 1	PEPBP1	P30086	187	NRPTSISWDGLDSGK	8.02
				LYEQLSGK	4.28
Syntaxin-1B	STX1B	P61266	288	QHSAILAAPNPDEK	4.86
Syntaxin-7	STX7	O15400	261	EFGSLPTTPSEQR	4.98
Complexin-2	CPLX2	Q6PUV4	134	AALEQPCEGSLTRPK	11.19
14-3-3 protein epsilon	YWHAE	P62258	255	IISSIEQK	3.14
14-3-3 protein eta	YWHAH	Q04917	246	AVTELNEPLSNEDR	5.69
14-3-3 protein zeta/delta	YWHAZ	P63104	245	VVSSIEQK	3.27
Gamma-synuclein	SNCG	O76070	127	ENVVQSVTSVAEK	10.24
Beta-synuclein	SNCB	Q16143	134	EGVVQGVASVAEK	9.70
Neurogranin	NRGN	Q92686	78	KGPGPGGPGGAGVAR	19.23
Neuronal pentraxin receptor	NPTXR	O95502	500	NNYMYAR	1.4
				LVEAFGGATK	2
Neuronal pentraxin-1	NPTX1	Q15818	432	ETVLQQK	1.62
				CESQSTLDPGAGEAR	3.47
				LTPGEVYNLATCSTK	3.47
Neuronal pentraxin-2	NPTX2	P47972	431	ETVVQQK	1.62
				VAELEDEK	1.86
**Endo-lysosomal panel**					
Cathepsin F	CTSF	Q9UBX1	484	TLLCSFQVLDELGR	2.89
				SDVPFWAIK	1.86
Ganglioside GM2 activator	GM2A	P17900	193	EVAGLWIK	4.15
				IESVLSSSGK	5.18
Lysosome-associated membrane glycoprotein 2	LAMP2	P13473	410	GILTVDELLAIR	2.93
				IPLNDLFR	1.95
AP-2 complex subunit beta	AP2B1	P63010	937	AVWLPAVK	0.85
				IQPGNPNYTLSLK	1.39

Supplementary Table 2: CSF Synaptic and endo-lysosomal biomarkers in samples collected in the evening vs morning.

Biomarkers	Morning concentration	Evening concentration	Differences morning-evening	% Change	F (df1, df2) [a]	P-value corrected (uncorrected) [b]
**Synaptic Panel**						
Amyloid Precursor Protein	3.80 (1.32)	4.04 (1.41)	0.24 (0.12 to 0.37)	7.06 (4.02 to 10.10)	15.46 (1, 37)	**0.005** (0.0004)
Neurosecretory protein VGF						
NSEPQDEGELFQGVDPR	3.77 (2.00)	3.98 (2.13)	0.21 (0.03 to 0.38)	6.38 (1.89 to 10.88)	5.84 (1, 37)	**0.041** (0.021)
AYQGVAAPFPK	7.52 (4.02)	8.08 (4.49)	0.56 (0.14 to 0.98)	8.45 (3.47 to 13.43)	7.41 (1, 37)	**0.023** (0.010)
Secretogranin-2						
ALEYIENLR	4.10 (1.55)	4.36 (1.74)	0.26 (0.06 to 0.47)	7.06 (2.65 to 11.47)	6.75 (1, 37)	**0.03** (0.013)
VLEYLNQEK	4.25 (1.66)	4.44 (1.83)	0.19 (-0.01 to 0.39)	5.08 (0.73 to 9.43)	3.74 (1, 37)	0.09 (0.06)
Chromogranin-A						
GLSAEPGWQAK	4.44 (2.84)	4.68 (3.23)	0.24 (0.01 to 0.47)	5.03 (1.05 to 9.01)	4.47 (1, 37)	0.07 (0.041)
EDSLEAGLPLQVR	4.04 (2.82)	4.38 (2.99)	0.34 (0.10 to 0.57)	9.38 (4.97 to 13.78)	8.2 (1, 37)	**0.02** (0.007)
Rab GDP dissociation inhibitor alpha	0.31 (0.09)	0.32 (0.09)	0.01 (-0.01 to 0.02)	2.67 (-1.26 to 6.60)	1.40 (1, 37)	0.31 (0.24)
Phosphatidylethanolamine-binding protein 1						
NRPTSISWDGLDSGK	7.91 (2.43)	8.23 (2.57)	0.32 (0.10 to 0.54)	4.45 (1.71 to 7.20)	8.96 (1, 37)	**0.02** (0.005)
LYEQLSGK	2.74 (0.77)	2.76 (0.73)	0.02 (-0.05 to 0.08)	1.17 (-1.24 to 3.58)	0.19 (1, 37)	0.68 (0.66)
AP-2 complex subunit beta						
AVWLPAVK	1.26 (0.46)	1.23 (0.44)	-0.03 (-0.07 to 0.01)	-1.60 (-4.53 to 1.32)	1.71 (1, 37)	0.27 (0.20)
IQPGNPNYTLSLK	1.61 (0.59)	1.58 (0.59)	-0.03 (-0.09 to 0.04)	-0.79 (-4.96 to 3.38)	0.63 (1, 37)	0.48(0.43)
Syntaxin-1B	0.31 (0.10)	0.34 (0.11)	0.02 (0.01 to 0.03)	7.10 (3.38 to 10.81)	13.77 (1, 37)	**0.005** (0.0007)
Syntaxin-7	0.30 (0.10)	0.31 (0.11)	0.01 (0.0002 to 0.02)	4.49 (0.87 to 8.12)	4.27 (1, 37)	0.07 (0.046)
Complexin-2	1.55 (0.53)	1.59 (0.52)	0.04 (-0.04 to 0.12)	4.17 (-0.71 to 9.05)	0.90 (1, 37)	0.42 (0.35)
14-3-3 epsilon	0.43 (0.14)	0.45 (0.16)	0.03 (0.008 to 0.05)	6.62 (2.29 to 10.96)	8.05 (1, 37)	**0.02** (0.007)
14-3-3 eta	0.21 (0.07)	0.22 (0.07)	0.005 (-0.01 to 0.02)	6.79 (-4.25 to 17.83)	0.58 (1, 37)	0.48 (0.45)
14-3-3 zeta/delta	1.25 (0.43)	1.26 (0.42)	0.01 (-0.04 to 0.06)	1.77 (-2.25 to 5.80)	0.12 (1, 37)	0.74 (0.73)
Gamma-synuclein	0.60 (0.21)	0.64 (0.20)	0.04 (0.006 to 0.07)	8.50 (3.31 to 13.70)	5.82 (1, 37)	**0.042** (0.021)
Beta-synuclein	0.28 (0.10)	0.30 (0.12)	0.03 (0.007 to 0.04)	9.59 (3.90 to 15.28)	7.91 (1, 37)	**0.02** (0.008)
Neurogranin	0.60 (0.28)	0.66 (0.31)	0.06 (0.02 to 0.1)	11.59 (6.64 to 16.54)	10.56 (1 ,37)	**0.012**(0.003)
Neuronal pentraxin receptor	6.17 (3.66)	6.34 (3.53)	0.18 (-0.10 to 0.46)	5.44 (1.11 to 9.76)	1.67 (1, 37)	0.27 (0.20)
NNYMYAR	1.22 (0.55)	1.35 (0.60)	0.12 (0.05 to 0.19)		13.58 (1, 37)	**0.005** (0.0007)

LVEAFGGATK				12.15 (6.15 to 18.15)		
Neuronal pentraxin-1						
ETVLQQK	0.67 (0.24)	0.71 (0.26)	0.04 (0.013 to 0.07)	7.66 (3.28 to 12.04)	8.38 (1, 37)	**0.02** (0.006)
CESQSTLDPGAGEAR	0.79 (0.40)	0.81 (0.40)	0.01 (-0.02 to 0.05)	1.46 (-3.34 to 6.26)	0.63 (1, 37)	0.48 (0.43)
LTPGEVYNLATCSTK	1.52 (0.46)	1.61 (0.49)	0.09 (0.04 to 0.14)	6.24 (3.02 to 9.47)	11.92 (1, 37)	**0.008** (0.001)
Neuronal pentraxin-2						
ETVVQQK	0.25 (0.11)	0.26 (0.12)	0.009 (-0.01 to 0.03)	7.25 (-2.64 to 17.14)	0.84 (1, 37)	0.43 (0.36)
VAELEDEK	1.43 (0.59)	1.50 (0.64)	0.07 (0.004 to 0.14)	5.98 (1.85 to 10.12)	4.61 (1, 37)	0.07 (0.039)
**Endo-lysosomal panel**						
Cathepsin F						
TLLCSFQVLDELGR	0.47 (0.11)	0.48 (0.11)	0.01 (-0.01 to 0.04)	3.97 (-0.64 to 8.58)	1.74 (1, 37)	0.27 (0.20)
SDVPFWAIK	0.45 (0.13)	0.49 (0.13)	0.04 (0.01 to 0.07)	13.29 (4.44 to 22.14)	7.97 (1, 37)	**0.02** (0.008)
Ganglioside GM2 activator						
EVAGLWIK	3.91 (1.45)	4.11 (1.49)	0.20 (0.01 to 0.30)	5.95 (2.94 to 8.96)	15.87 (1, 37)	**0.005** (0.0003)
IESVLSSSGK	5.38 (1.95)	5.60 (2.01)	0.23 (0.11 to 0.35)	4.70 (2.27 to 7.13)	14.15 (1, 37)	**0.005** (0.0006)
Lysosome-associated membrane glycoprotein -2						
GILTVDELLAIR	22.2 (8.73)	23.9 (9.56)	1.66 (0.20 to3.11)	10.76 (3.18 to 18.34)	5.33 (1, 37)	0.051 (0.027)
IPLNDLFR	22.5 (9.97)	23.4 (9.21)	0.89 (-0.06 to1.84)	5.69 (2.03 to 9.35)	3.64 (1, 37)	0.09 (0.06)
AP-2 complex subunit beta						
AVWLPAVK	1.26 (0.46)	1.23 (0.44)	-0.03 (-0.07 to 0.01)	-1.60 (-4.53 to 1.32)	1.71 (1, 37)	0.27 (0.20)
IQPGNPNYTLSLK	1.61 (0.59)	1.58 (0.59)	-0.03 (-0.09 to 0.04)	-0.79 (-4.96 to 3.38)	0.63 (1, 37)	0.48(0.43)

Data shown as mean (SD) or mean (95%CI) unless specified otherwise. Abbreviations: CSF, cerebrospinal fluid.

[a] Main effects of sampling time from repeated measures two-way ANOVA

[b] P-values were corrected for multiple comparison using the FDR method

Supplementary Figure 1: CSF synaptic and endo-lysosomal biomarkers concentrations with increased levels in samples collected in the morning compared to those collected in the evening.

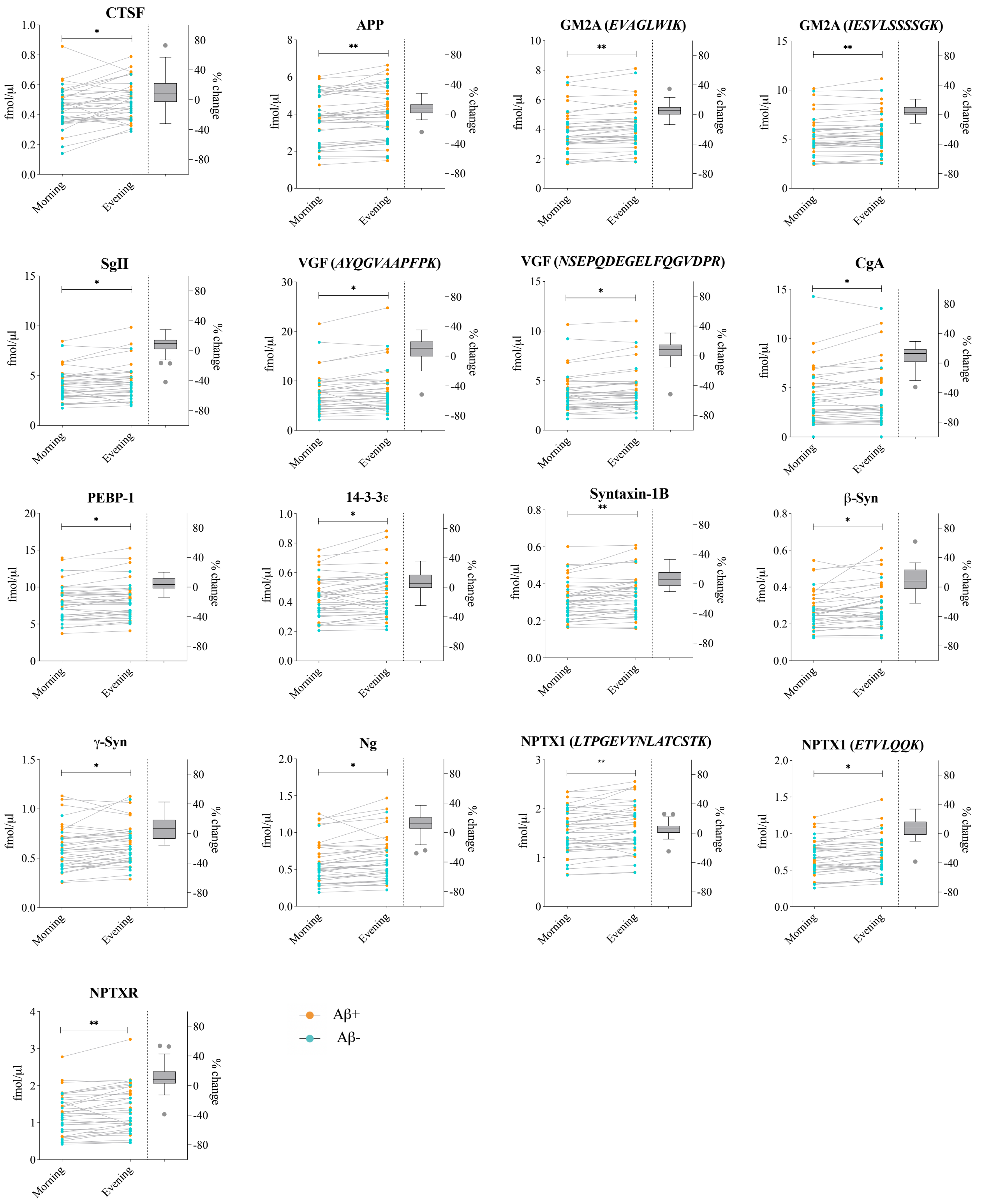

Subject specific biomarker concentration in samples collected in the morning vs evening. Average percent changes between time points are shown in box-plots plotted with the Tukey method. Blue and orange dots represent participants with negative and positive amyloid status, respectively. Asterisks represent p-values for the main effects of sampling time from repeated measures two-way ANOVA; * p<0.05, **p<0.01

Supplementary Table 3: The effects of interaction between sampling time and amyloid status or sampling time and sampling order on the core AD biomarkers. Data are F and p-values for the interaction between sampling time and amyloid status or sampling time and sampling order from repeated measures two-way ANOVA.

	Amyloid Status Sampling time*		*Sampling order* Sampling time*	
	**F(df,df)**	**p-value[a]**	**F(df,df)**	**p-value[a]**
CSF Aβ42	0.75 (1,36)	0.39	1.321 (1,36)	0.26
CSF Aβ40	1.66 (1,36)	0.21	0.04 (1,36)	0.85
CSF Aβ42/40	2.55 (1,36)	0.12	2.44 (1,36)	0.13
CSF P-tau217	3.07 (1,36)	0.09	1.02 (1,36)	0.32
CSF P-tau23b[a]	3.70 (1,35)	0.06	0.11 (1,35)	0.74
CSF P-tau18b[a]	3.44 (1,35)	0.07	0.95 (1,35)	0.34
CSF GFAP	0.22 (1,36)	0.64	2.96 (1,36)	0.09
CSF NfL	0.13 (1,36)	0.72	0.35 (1,36)	0.56
CSF t-tau	8.13 (1,36)	0.007	0.06 (1,36)	0.81
Plasma Aβ42	0.02 (1,36)	0.88	0.15 (1,36)	0.70
Plasma Aβ40	0.59 (1,36)	0.45	1.02 (1,36)	0.32
Plasma Aβ42/40	0.91 (1,36)	0.35	1.68 (1,36)	0.20
Plasma P-tau217	1.30 (1,36)	0.26	1.17 (1,36)	0.29
Plasma P-tau231[c]	0.47 (1,34)	0.50	0.70 (1,34)	0.41
Plasma P-tau181	0.05 (1,36)	0.82	0.17 (1,36)	0.68
Plasma GFAP	1.28 (1,36)	0.27	4.08 (1,36)	0.051
Plasma NfL	0.63 (1,36)	0.43	1.321 (1,36)	0.26

Abbreviations: CSF, cerebrospinal fluid; Aβ, amyloid beta; GFAP, glial fibrillar acidic protein; NfL, neurofilament light; t-tau, total tau; P-tau, phosphorylated tau. [a] Uncorrected p-values, [b] Data was missing for 1 participant; [c] Data was missing for 2 participants.

Supplementary Figure 2: CSF biomarkers levels in samples collected in the morning and evening separated by amyloid status

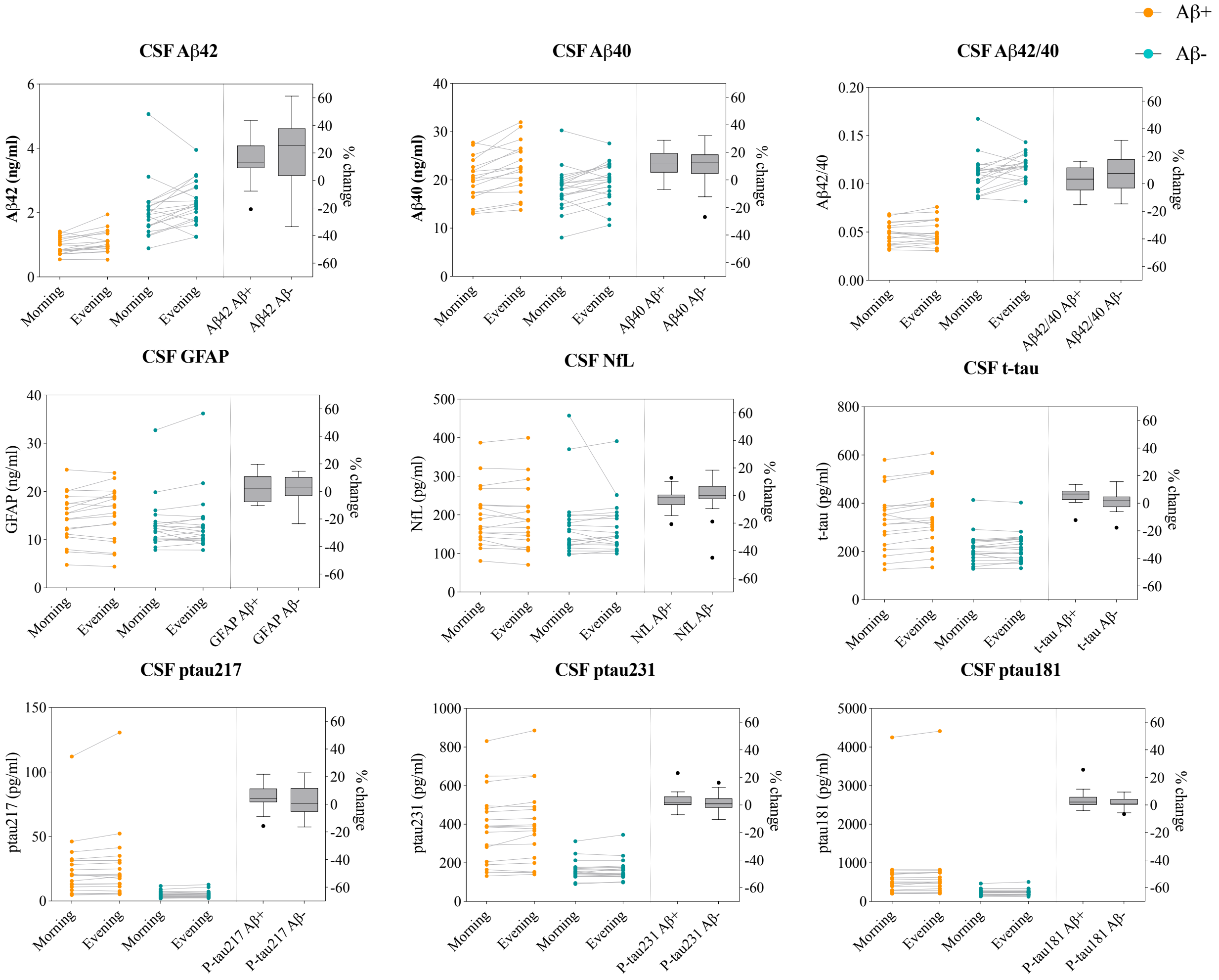

Subject specific biomarker concentration in samples collected in the morning vs evening. Average percent changes between time points are shown in box-plots plotted with the Tukey method. Blue and orange dots represent participants with negative and positive amyloid status, respectively.

Supplementary Figure 3: Plasma biomarkers levels in samples collected in the morning and evening separated by amyloid status

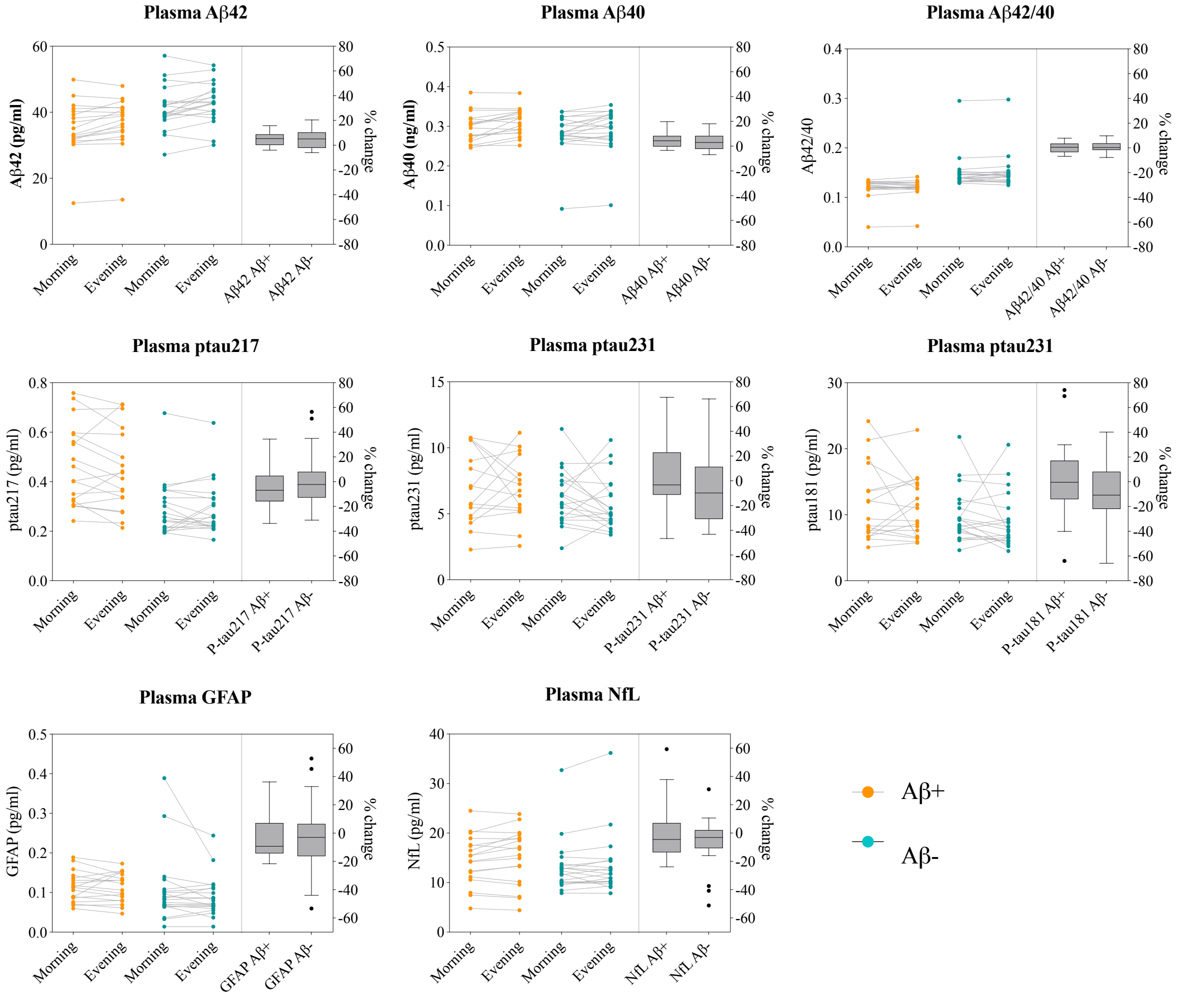

Subject specific biomarker concentration in samples collected in the morning vs evening. Average percent changes between time points are shown in box-plots plotted with the Tukey method. Blue and orange dots represent participants with negative and positive amyloid status, respectively.
